# The complete chloroplast genome sequence of a traditional Chinese medicine plant *Bulbophyllum disciflorum* Rolfe (Orchidaceae)

**DOI:** 10.1080/23802359.2019.1670112

**Published:** 2019-12-09

**Authors:** Yang Tang, Jiapeng Yang, Zhitao Niu, Xiaoyu Ding

**Affiliations:** a College of Life Sciences, Nanjing Normal University, Nanjing, China;; b Jiangsu Provincial Engineering Research Center for Technical Industrialization for Dendrobium, Nanjing, China

**Keywords:** *Bulbophyllum disciflorum*, traditional Chinese medicine, chloroplast genome, Epidendroideae

## Abstract

*Bulbophyllum disciflorum* is one of Orchidaceae species, which has important ornamental and economic value. Here, we reported the first chloroplast genome sequence of *Bulbophyllum*. The genome of *B. disciflorum* is 148,554 bp in length, including a large single-copy (LSC) of 79,001 bp, a small single-copy (SSC) of 16,797 bp, and a pair of inverted repeat regions (IR_a_ and IR_b_) of 26,378bp. It contains 108 unique genes consisting of 74 protein-coding genes, 30 tRNA genes, and 4 rRNA genes. The phylogenetic analysis revealed that *Bulbophyllum* is sister to the genus of *Dendrobium*.

The genus *Bulbophyllum*, one of the largest genera in the family Orchidaceae, comprising approximately 2200 species, mainly distributed in tropical Asia, America, Africa, and Australasia. In China, there are about 110 species of this genus, some of which have been extensively used as Traditional Chinese Medicine (TCM) for hundreds of years. However, many wild *Bulbophyllum* species are in extreme danger of extinction. Here, we sequenced and assembled the complete chloroplast genome sequence of *B*. *disciflorum*, which is the first one in the genus, and reconstructed the phylogenetic relationship with other Orchidaceae species. Such a chloroplast genome sequence could provide abundant genetic information for identification, utilization, and breeding of *Bulbophyllum* species.

For the DNA extraction of *B. disciflorum*, 2 g of fresh leaves were harvested. The plant was stored in the College of Life Sciences, Nanjing Normal University (voucher specimen No. Niu19015). Total genomic DNA was extracted using the DNeasy Plant Mini Kit (Qiagen, Dusseldorf, Germany) according to the manufacturer's instructions. Approximately, 3.5 Gb of 150 bp pair-end reads were yielded using an Illumina Hiseq4000 sequencer, and assembled on SOAP de novo (v2.04) and CLC Genomics Workbench 8.5.1 (CLC Bio, Aarhus, Denmark) as previously reported (Niu et al. 2017). The gaps and junctions between inverted repeat (IR) regions and single copy (SC) regions were confirmed by PCR amplification. Genes were annotated using DOGMA (Wyman et al. 2004) and tRNAscan-SE 1.21 (Schattner et al. 2005). The new annotated chloroplast genome sequence was deposited in GenBank (Accession No. LC498826).

The newly sequenced chloroplast genome of *B. disciflorum* was 148,554 bp long with GC content of 37.94%. The genome consisted of an LSC region of 79,001 bp, an SSC region of 16,797 bp, and a pair of IR regions of 26,378 bp each. The chloroplast genome of *B. disciflorum* was well conserved, containing 30 unique tRNA genes, four unique rRNA genes, and 68 unique protein-coding genes (Figure 1). The sequence of eleven plastid NDH genes was compared to *Apostasia odorata* (NC_030722) which contains full set of functional NDH genes in orchids. Like other orchid species, *B. disciflorum* also experienced the loss of NDH genes. Among them, only *the* genes in IR regions contain full reading frames, whereas other ten plastid NDH genes were truncated or completely lost.

**Figure 1.**
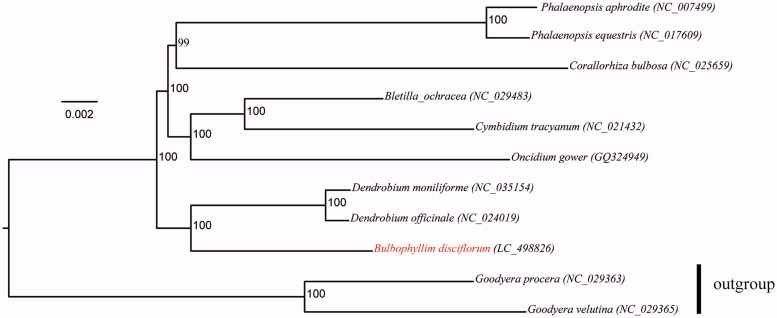
Maximum likelihood tree of Epidendroideae species based on the whole chloroplast genome sequences with *Goodyera procera* and *Goodyera velutina* as outgroup. Numbers near the nodes represent ML bootstrap values.

The phylogeny of Epidendroideae was reconstructed based on the multiple alignments of reported 11 orchid chloroplast genomes with *Goodyera procera* (NC_029363) and *Goodyera velutina* (NC_029365) as outgroup using MAFFT v. 7.427 (Nakamura et al. 2018). The best-fitting model of nucleotide substitution was GTR + I+G, as determined by the Akaike Information Criterion (AIC) in jModelTest v. 2.1.7 (Darriba et al. 2012). Maximum Likelihood (ML) analysis was conducted using RAxML 8.0.2 with 100 bootstrap replicates (Stamatakis 2014). As shown in Figure 1, the ML tree revealed: (1) the species of Epidendroideae formed a monophyly group; (2) the sister relationship between the genus of *Bulbophyllum* and *Dendrobium* was strongly supported (BS value = 100). These results were congruent with that revealed in the previous studies based on cpDNA fragments (Luo et al. 2014).
